# Climate drivers of vector-borne diseases in Africa and their relevance to control programmes

**DOI:** 10.1186/s40249-018-0460-1

**Published:** 2018-08-10

**Authors:** Madeleine C. Thomson, Ángel G. Muñoz, Remi Cousin, Joy Shumake-Guillemot

**Affiliations:** 1 International Research Institute for Climate and Society (IRI), Earth Institute, Columbia University, New York, USA; 2 Mailman School of Public Health Department of Environmental Health Sciences, Columbia University, New York, USA; 3 IRI-World Health Organization (WHO) Collaborating Centre (US 430) on Early Warning Systems for Malaria and other Climate Sensitive Diseases, New York, USA; 4 Atmospheric and Oceanic Sciences, Princeton University, Princeton, NJ USA; 5 World Health Organization- World Meteorological Organization Joint Climate and Health Office, WMO, Geneva, Switzerland; 6 International Research Institute for Climate and Society, LDEO, Palisades, New York, 10964 USA

**Keywords:** Vector-borne diseases, Climate variability, Climate change, El Niño southern oscillation, Climate services, Adaptation, Africa

## Abstract

**Background:**

Climate-based disease forecasting has been proposed as a potential tool in climate change adaptation for the health sector. Here we explore the relevance of climate data, drivers and predictions for vector-borne disease control efforts in Africa.

**Methods:**

Using data from a number of sources we explore rainfall and temperature across the African continent, from seasonality to variability at annual, multi-decadal and timescales consistent with climate change. We give particular attention to three regions defined as WHO-TDR study zones in Western, Eastern and Southern Africa. Our analyses include 1) time scale decomposition to establish the relative importance of year-to-year, decadal and long term trends in rainfall and temperature; 2) the impact of the El Niño Southern Oscillation (ENSO) on rainfall and temperature at the Pan African scale; 3) the impact of ENSO on the climate of Tanzania using high resolution climate products and 4) the potential predictability of the climate in different regions and seasons using Generalized Relative Operating Characteristics. We use these analyses to review the relevance of climate forecasts for applications in vector borne disease control across the continent.

**Results:**

Timescale decomposition revealed long term warming in all three regions of Africa – at the level of 0.1–0.3 °C per decade. Decadal variations in rainfall were apparent in all regions and particularly pronounced in the Sahel and during the East African long rains (March–May). Year-to-year variability in both rainfall and temperature, in part associated with ENSO, were the dominant signal for climate variations on any timescale. Observed climate data and seasonal climate forecasts were identified as the most relevant sources of climate information for use in early warning systems for vector-borne diseases but the latter varied in skill by region and season.

**Conclusions:**

Adaptation to the vector-borne disease risks of climate variability and change is a priority for government and civil society in African countries. Understanding rainfall and temperature variations and trends at multiple timescales and their potential predictability is a necessary first step in the incorporation of relevant climate information into vector-borne disease control decision-making.

**Electronic supplementary material:**

The online version of this article (10.1186/s40249-018-0460-1) contains supplementary material, which is available to authorized users.

## Multilingual abstracts

Please see Additional file 1 for translations of the abstract into the six official working languages of the United Nations.

## Background

### Climate and vector borne disease

Many parasitic, viral, and bacterial diseases respond to variations in the climate whether through their geographic distribution, seasonality, inter-annual variability, or temporal and spatial trends. Detailed reviews of climate variables and the impact on pathogen and vector dynamics are available for a wide range of diseases [1, 2].

Known relationships of climate variability and change and the climate-sensitivity of most important infectious diseases causing considerable morbidity and mortality worldwide suggests the potential role of climate information in improving climate sensitive health outcomes [3]. Although many infectious diseases of humans are climate sensitive – those that are transmitted by arthropod (insect and tick) and snail vectors are particular important in lower and middle income countries [4]. They are therefore prioritized by the Tropical Disease Research [5] initiative of the World Health Organization and partners [5, 6].

#### Impact of climate on vector-borne disease transmission dynamics

Weather and climate conditions, as well as surface water availability, that can influence positively or negatively the transmission of arthropod-borne diseases include air and water temperature, rainfall, humidity, surface water and wind [7]. These conditions, may also manifest as extreme events causing flooding, drought, storms and heat/cold waves – impacting directly and indirectly on vector transmission dynamics. The direct impacts of climate on disease vectors are via adult survival and reproduction rates, the creation of breeding sites, and the development rates of the juvenile stage of the vector [8]. Pathogens transmitted to humans by insects and ticks spend part of their life cycle in their cold-blooded secondary (non-human) host where they are effectively at the temperature of the local micro-climate. Here the development rate of the pathogen (called the extrinsic incubation period) will slow down at lower temperatures increasing the probability that the insect/tick will not survive long enough for disease transmission to occur. Some interactions between vector/parasite and climate are relatively simple to model (e.g. the relationship between rainfall and breeding sites) but others are complex. For example, temperature interacts in multiple, sometimes opposing ways with different aspects of insect or pathogen biology. Despite this complexity, it is clear that, to varying degrees, climatic factors determine the geographic limitations of vector-borne diseases, their seasonal occurrence, year to year variability as well as medium and long term shifts in both geographic distribution and intensity of transmission.

In Africa, rainfall, humidity and temperature are major constraint to the development of vegetation, soils, water sources, agriculture and therefore the livelihoods of the continents diverse populations [9]. Understanding the spatial and temporal relationships of climate and environmental direct and indirect drivers of vector-borne disease transmission is important in order to benefit from climate information to better target current control activities or predict future challenges.

#### Temporal lags in observed climate and vector-borne diseases

The temporal dynamics of diseases transmitted by insects and ticks will lag factors such as rainfall, temperature and humidity by a number of months because of the many inbuilt delays to the transmission process [10]. For example, rainfall creates potential breeding sites for juvenile mosquito vectors, but newly laid eggs need time to mature as larvae and pupae before they emerge as adult mosquitoes capable of transmitting disease [11]. After emergence, the adult female mosquito needs to imbibe the pathogen (e.g. malaria parasite or dengue virus) from an infectious human host before transmitting it, after it completes its extrinsic incubation period, to another person [11]. In epidemic prone regions (such as semi-arid areas or highland areas bordering endemic zones), infection and immunity in the human host population are low at the beginning of the epidemic wave and therefore a number of blood meals, each separated by the days needed to complete the gonotrophic cycle, may be needed before a female mosquito encounters and infectious human host [11]. Further delays in the development of an epidemic result from the time taken between the human host being infected and being infectious – a process that takes place at the more or less consistent temperature of the human host. The result of these lags is that cumulative observed weather events and/or conditions may provide approximately 2–4 months warning of vector-borne disease outbreaks depending on local circumstances. Shorter lags usually occur in warmer environments where development rates of vector and parasite are faster. However warmer environments may be associated with drought which will likely (but not always) reduce vector breeding sites and adult mosquito survivorship. Understanding how climate drives disease transmission in a particular locale is a step towards using climate information to control disease [4].

#### Development of early warning systems (EWS)

If significant temporal relationships between the occurrence of specific climatic/environmental variables and human cases of vector-borne diseases are demonstrated, and an underlying mechanism is understood, then it is possible to consider the development of a climate-informed early warning systems [12]. EWS may help disease control services anticipate where and when outbreaks or increased transmission are likely to occur and react proactively to emerging changes in disease risk.

Disease early warning systems may be established based on epidemiological data alone. For instance, an unusual early seasonal rise in case numbers may trigger an epidemic alert for some diseases. These are often called “early detection systems” but in reality they are still providing early warning of likely increase in future cases [13]. Early warning can be extended using observed environmental or climatic data which may offer 2–3 months prior notice of likely changes in transmission risk. Early warning for climate sensitive diseases can be further extended by 3–6 months using seasonal climate forecasts [14].

Weather forecasts (< 2 weeks), on the other hand add little value to the prediction of a vector-borne disease epidemics. This is because they provide only a few additional days to early warning system that already have the potential for several months lead time just using observed climate or environmental data alone.

Sub-seasonal to seasonal (termed S2S) forecasts are currently an intense area of climate and weather research and may, in the future, provide additional predictability at the two week to two month time frame. Because of the short prediction time frame in Africa of weather forecasts (1–5 days) and the experimental nature of S2S forecasts neither are considered further here. However, as the science advances, opportunities for using S2S forecasts in vector disease control programmes may emerge.

Decadal (10–30 year) and long-term shifts in the climate may also impact on vector-borne diseases by changing their geographic range. In a recent study of warming in the East African highlands the authors calculated that an additional 6 million individuals now live in regions of Ethiopia that are above the temperature threshold for malaria transmission compared with 30 years ago; this change resulting from a slow upward shift in minimum temperature [15]. However, while decadal variations in the climate are increasingly understood to exist, our ability to predict such changes in an operational context is not currently developed and may yet prove impossible because of the strong stochastic character of the climate [16]. Trends in temperature, where decadal variations are weak, provide an indication of longer term climate changes.

The climate information regarding climate change timescale (> 50 years) are highly uncertain and beyond the normal decision timeframe of Ministries of health; they are considered here in the context of historical trends.

### The African climate system and its drivers at multiple time-scales

**Fig. 1:**
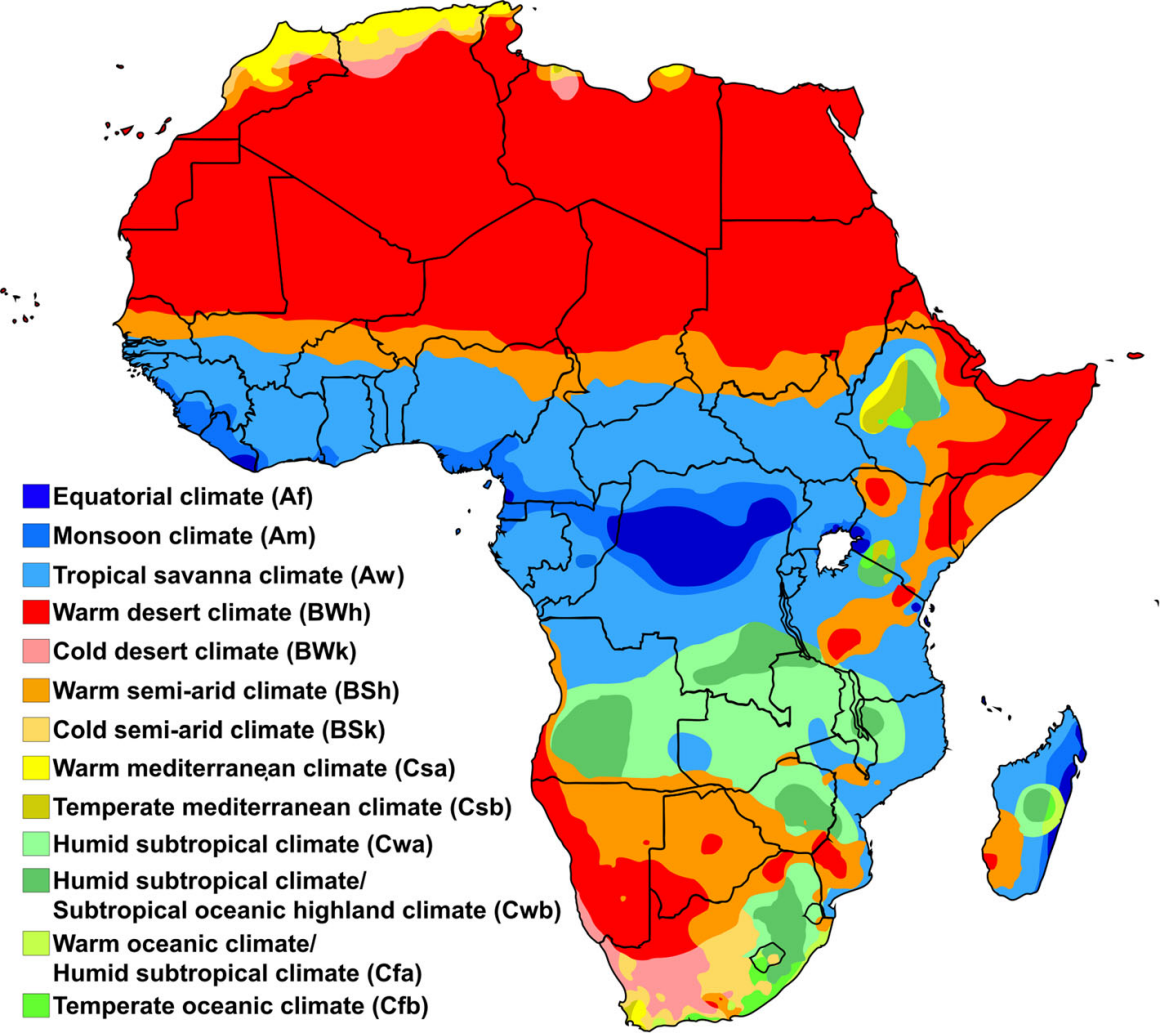
Koppen-Geiger climate classification scheme for Africa [12]

**Fig. 2:**
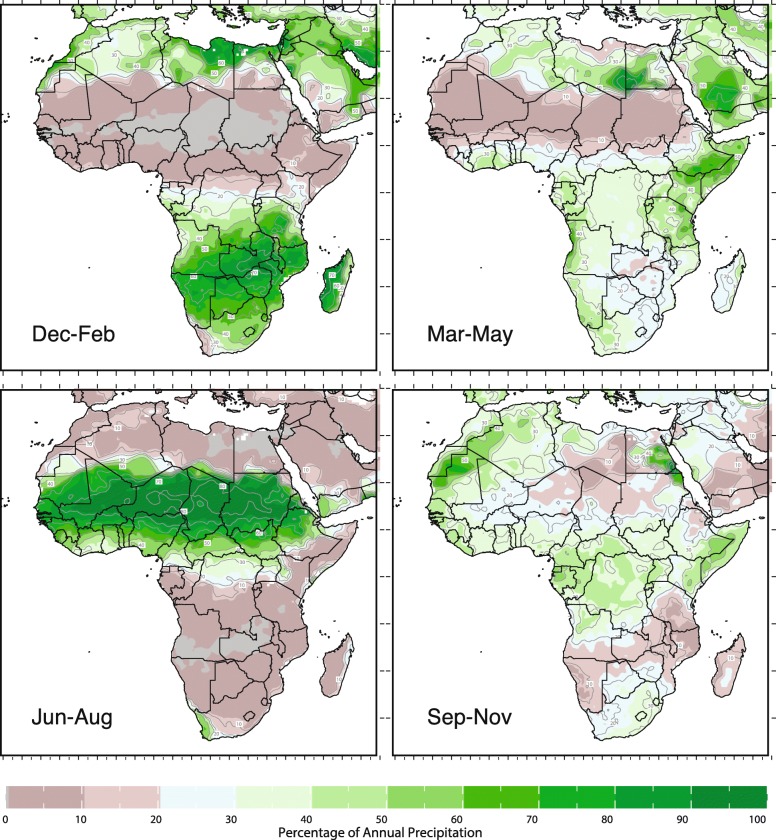
Percentage of mean seasonal rainfall for Dec–Feb, Mar–May, Jun–Aug, and Sep–Nov. Data from the Global Precipitation Climatology Centre, 1971–2000

The health and wellbeing of African populations is closely tied to their environment which is itself closely linked to the regional and local climate. An extreme range of climates span the continent, according to the Köppen-Geiger classification system (Fig. 1) [17]. Across the continent the climate varies from arid zones (including the Sahara, Somali-Chalbi and Kalahari deserts), steppe or semi-arid regions (e.g. Sahelian savannah) to humid tropical environments (Congo river basin). Humid subtropical climates are features found predominantly in southern Africa but also include areas in the Ethiopian highlands. In some regions these widely diverse climates co-exist within relatively small areas and rainfall amount and seasonality (for example) may change significantly over tens of kilometers [18]. The changes in seasons (particularly the rainy and dry seasons) is the dominant characteristic of regional climate and it consequently drives the seasonal pattern of human activities as well as vector-borne diseases across the continent. The large seasonal variations in rainfall that distinguish different climate zones is seen clearly in Fig. 2a–d –which indicates the fraction of mean annual rainfall that falls within 3 month seasons (December–February: DJF; March–May: MAM; June–August: JJA; September–November: SON). The Fig. 2b and d indicate that East Africa has a bimodal season while others, such as the Sahel (see Fig. 2c) have a single rainy season, more typical of monsoon behavior.

The most significant driver of seasonal temperature change across Africa (where proximity to the equator might suggest nearly constant year-round temperatures) is the monsoonal rains, in part related to the inter-tropical convergence zone defined previously. For instance, cloud cover at night will tend to increase minimum temperatures whereas cloud cover in the day time will tend to reduce maximum temperatures [19]. These different responses indicate that minimum and maximum temperatures are better treated as separate variables rather than combined as mean temperature.

Whereas weather is almost entirely governed by conditions in the atmosphere, the climate is substantially driven by slower processes, particularly in the major oceans. The climate at any location varies from its mean historical climate state on multiple time-scales, from annual to multi-decadal (10–30 years) to long-term climate change; the latter compatible with anthropogenic climate change signals. The magnitude of these variations and trends may enhance or decrease the climate suitability for different disease vectors and their pathogens.

**Fig. 3:**
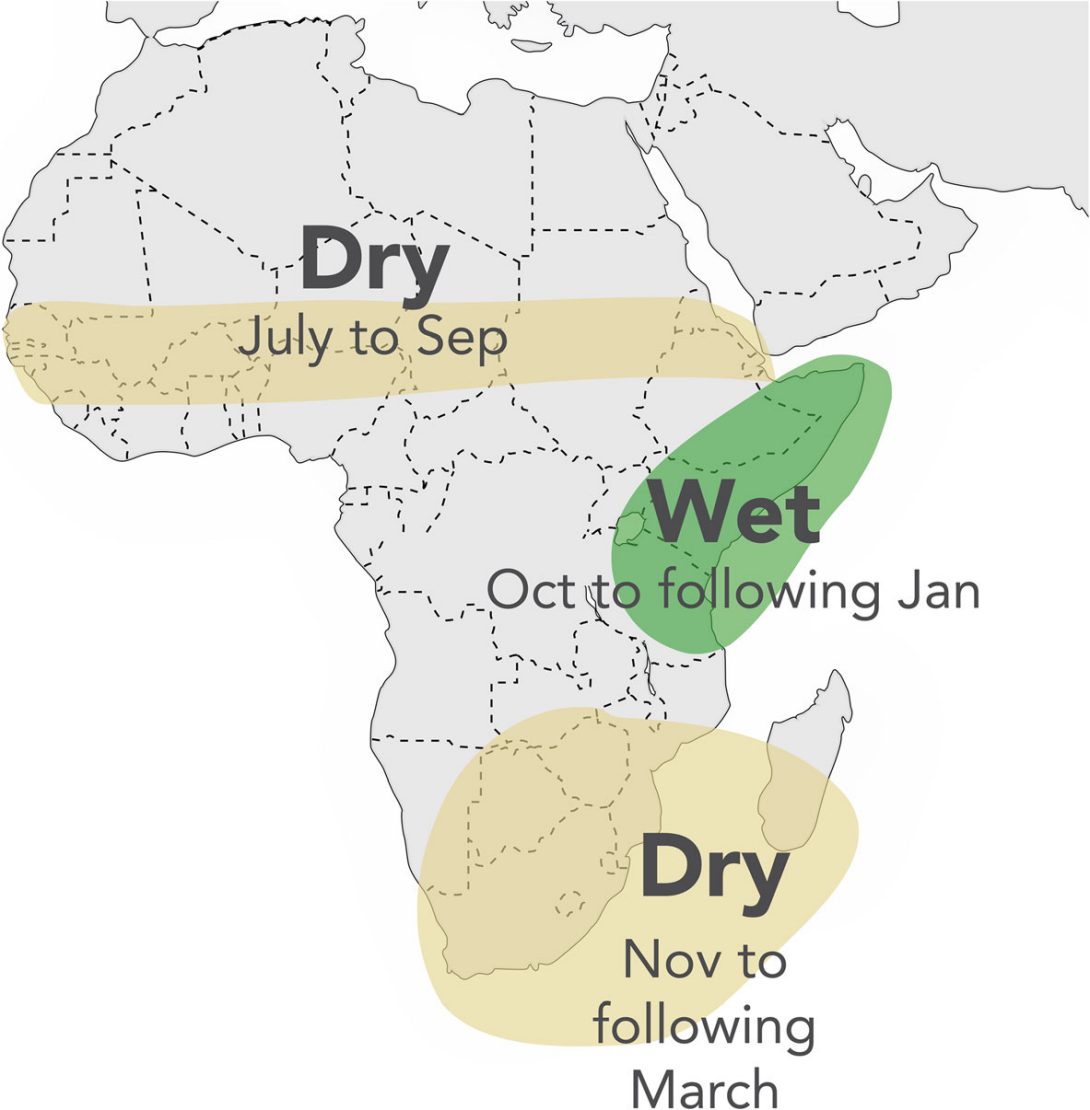
Likely impact of El Niño rainfall in Africa. In addition, general warming of the atmosphere occurs across the tropics during an El Niño event. Local temperature will be influenced by rainfall

Sea surface temperature variations in the Atlantic [20], Indian [21] and Pacific [22] oceans influence the African climate on different time scales. We consider three timescales of variability in the African climate that describe the past and provide some indication of the future. El Niño-Southern Oscillation (ENSO) is the most important driver of climate variability at seasonal-to-interannual timescales [23], a key source of climate predictability in Africa [24] (see Fig. 3) and relevant to the development of climate information services targeting health decision-makers [3]. It is important to recognize that ENSO (El Niño and La Niña) impact the climate (and thereby climate-sensitive health outcomes): (a) differently according to the variable of interest (e.g. rainfall, and minimum and maximum temperature), (b) at different spatial scales, (c) in some regions and not others, (d) in some seasons and not others, (e) often according to its strength, and sometimes in a non-linear fashion, (f) at varying periods (from 5 months to ~ two years), with both El Niño and La Niña events on occasions occurring in the same calendar year (e.g., 2010), (g) often substantially conditioned on the action of other climate drivers, such as the Indian Ocean Dipole [25].

Natural variations in the climate at 10–30 year time frames (decadal) have also been observed in Western, Eastern and Southern Africa and again may be specific to region and season. In Eastern Africa decadal rainfall variations are largely confined to the long rains which occur between March and May [26]. Where historical data is sufficient, long term trends in temperature and rainfall, consistent with climate change, may be established once the noise from shorter term natural variations in the climate have been removed. Unless the impact of the different timescales can be disentangled, there is considerable opportunity for confusion, with important implications for decision-making and potential maladaptation. For instance, climate change models have indicated that Eastern Africa will become wetter towards the end of the twenty-first century while the region has, since 1999, experienced an increased frequency of drought [27].

Here we aim to characterize the African climate – its variability, trends and potential predictability – and establish the relevance of this knowledge and current tools to operational vector-borne disease control efforts.

## Methods

We use a range of data sources and analytical methods to undertake four analysis which we use to characterize the African climate and its potential predictability.

First we use global climate products to explore the nature of rainfall and temperature at multiple timescales (seasonal, decadal and long term change) in three regions in Africa. The regions chosen correspond to those used by the World Health Organization (WHO)-Special Programme for Research and Training in Tropical Diseases (TDR) “*Population Health Vulnerabilities to Vector-Borne Diseases: Assessing and Developing Strategies for Reducing the Impact of Social, Environmental and Climate Change in Africa*” research consortium partners [6]. These are: West Africa (Ivory Coast and Mauritania), East Africa (Kenya and Tanzania) and Southern Africa (Botswana, Zimbabwe). We then use global climate products to Identify regions and seasons across Africa where the ENSO has greatest impact on local temperature and rainfall. We then repeat the same analysis using climate products created through the “Enhancing National Climate Services (ENACTS)” initiative [28] for Tanzania and identify where ENSO has the greatest likely impact. Global climate products provide an assessment of where and when seasonal climate forecasts may be relevant to vector control efforts across the African continent.

### International research Institute for Climate and Society (IRI) data library

The IRI Data Library [29] was used throughout this study to access, manage, and analyse climate data as well as to display the results via Maprooms all of which are available to the reader (Table 1). The Data Library is an open and free earth science data service, providing common, high-quality, objective observations and analysis of the environment that promotes transparency in data source and manipulation. The platform makes climate and other data products more widely accessible through tool development, data organization and transformation, as well as data/technology transfer [30]. Tools developed include Maprooms which are designed for rapid access to needed information for particular user groups. Data Library technology has been shared with partners around the world and underpins key climate services in many countries including those implementing ENACTS initiative in Africa [28].

**Table 1:** IRI Data Library Maprooms used in the analysis

Maproom	
Timescale decomposition	https://iridl.ldeo.columbia.edu/maproom/Global/Time_Scales/
ENSO rainfall	https://iridl.ldeo.columbia.edu/maproom/Health/Regional/Africa/Malaria/ENSO_Prob/ENSO_Prob_Precip.html
ENSO temperature	https://iridl.ldeo.columbia.edu/maproom/Health/Regional/Africa/Malaria/ENSO_Prob/ENSO_Prob_Temp.html
Predictability of climate	http://iri.columbia.edu/our-expertise/climate/forecasts/seasonal-climate-forecasts/
ENACTS all countries	Iri.columbia.edu/ENACTS
ENACTS Tanzania	http://maproom.meteo.go.tz/maproom/

### Analysis 1. Multi-timescale climate decomposition

To better understand how much of the total variance in rainfall and temperature anomalies across the African continent is explained by different time-scales, a ‘timescale decomposition’ methodology [31] was used. The temporal analysis was focused on the WHO-TDR study sites. This approach has been used elsewhere to explore the contribution of climate variations and trends at multiple timescales to the observed seasonal climate of Latin America associated with the 2015 Zika virus epidemic [32].

#### Data

Timescale decomposition analysis was undertaken using the most up-to-date long-term rainfall and average temperature data available from the University of East Anglia’s Climate Research Unit, gridded station product version 3.4 (CRUv3.4, 0.5° resolution) [33], considering the period 1901–2000. It is widely recognized that changes in the number of observing station data incorporated into the monthly gridded data sets may significantly affect the results of any analysis. There has been a notable decline in stations available for incorporation into global products post 2000, so the analysis is limited to the twentieth century data only.

#### Methodology

The timescale decomposition methodology filters the associated anomalies of a climate time-series into three components: the inter-annual (year to year), decadal (10–30 year), and long-term trend signals. Time series, maps and data are freely available in the IRI’s Timescale Decomposition Maproom (https://iridl.ldeo.columbia.edu/maproom/Global/Time_Scales/) for any region in the world with long enough quality-controlled records. Data processing consists of three steps: (1) *Screening* the individual gridboxes for filled rainfall or temperature values, and for very dry regions and seasons; (2) *detrending* in order to extract slow, trend-like changes; and (3) *filtering*, to separate high and low frequency components in the detrended data.

### Analysis 2: Assessing the impact of the ENSO on rainfall and temperature across Africa

In Africa ENSO impacts on African rainfall are well known and vary according to region and season [24]. While the impact of ENSO on global tropical temperatures is also widely appreciated [34], local effects are amplified or muted by ENSO impacts on rainfall [19]. The rainfall response to ENSO is nearly contemporaneous however, this may not be true for temperature. Once El Niño has begun, there is a ramp up of global temperatures which are then slow to dissipate after the return to a neutral phase although they may cool down rapidly if La Niña conditions emerge.

#### Data

For sea-surface temperature (SST) data, the extended reconstructed SST (ERSST) dataset (http://iridl.ldeo.columbia.edu/SOURCES/.NOAA/.NCDC/.ERSST/.version4/.sst/) was used. The ENSO state for each season was defined according to the Oceanic Niño Index (ONI) [35]. This is calculated using SST anomalies based on the 1981–2010 normal, in the geographical box defined by 170°W, 5°S, 120°W, 5°N. A season is considered El Niño (La Niña) if it is part of at least 5 consecutive overlapping 3-month long seasons where the ONI is above 0.45 °C (below–0.45 °C).

Rainfall and temperature data correspond to the University of California Santa Barbara CHIRPS v2p0 monthly global precipitation, and the East Anglia University Climate Research Unit (http://iridl.ldeo.columbia.edu/SOURCES/.UCSB/.CHIRPS/.v2p0/.monthly/.global/.precipitation/).

TS3.23 near-surface temperature on a 0.5° × 0.5° lat/long grid (about 50 km of resolution) (http://iridl.ldeo.columbia.edu/SOURCES/.UEA/.CRU/.TS3p23/.monthly/.tmp/).

#### Methodology

The historical probability of seasonal average rainfall falling within the upper (wet/hot), middle (normal), or bottom (dry/cool) one-third (“tercile”) of the 1981–current historical distribution in Africa given the state of ENSO (El Niño, Neutral, La Niña) during that same season was calculated and the results presented in an IRI Maproom. The seasonal skill was assessed using the Generalized Relative Operating Characteristics (GROC), a metric similar to Kendall’s t rank correlation coefficient [36] measuring the “proportion of all available pairs of observation of differing category whose probability forecasts are discriminated in the correct direction” [37]. Being a discrimination metric, GROC provides information about how well the forecast system can distinguish between the different categories, e.g., above-normal from normal rainfall. It also provides an indication of how often the forecasts are correct, with a value of 50% (or 0.5) being the expected score of an unskilled set of forecasts [36].

### Analysis 3: Assessing the local impact of ENSO on rainfall and temperature in Tanzania

The analysis for one of the WHO-TDR study sites Monduli, Arusha, Tanzania – was further investigated using products and services from the ENACTS initiative [28]. ENACTS national climate products (rainfall and temperature) are created by quality – controlling all national station observations and combining this data with data from proxies – satellite estimates for rainfall, digital elevation models, and reanalysis products for temperature. The approach thus combines the spatial information from the proxies with the accuracy from point station measurements. The final products are datasets with 30 or more years of rainfall and temperature time-series data at a ten-daily (dekadal) time scale for a 4-km grid across the country. ENACTS products and services are disseminated online via Maprooms that are developed using the lRI Data Library which is installed at the Tanzanian Meteorological Agency [30] as well as in a number of other African countries (iri.columbia.edu/resources/ENACTS). This online mapping service provides user-friendly tools for the analysis, visualization, and download of climate information products via the NMHS websites.

#### Data

For ENSO the NOAA NCDC ERSST (version 4) was used when analyzing SSTs was used. For climate the ENACTS historical rainfall and temperature (minimum) databases (1983–2014) generated from combining quality-controlled station observations with satellite data and downscaled reanalysis data respectively were used.

#### Methodology

The approach used was the same as that undertaken for assessing the impact of the ENSO on rainfall and temperature across Africa (Analysis 2).

### Analysis 4: Assessment of seasonal rainfall and temperature predictability across Africa

Having identified the dominant signals of rainfall and temperature variability and trends in the different regions of the African continent, we explore their predictability using a two-tiered atmospheric global circulation model forecast system based on sea surface temperatures.

#### Data

The gridded global Climate Anomaly Monitoring System dataset from National Oceanic and Atmospheric Administration (NOAA) [12] is used for temperature. For precipitation, two datasets are used, depending on the period of interest: from 1979 onward the dataset is the Climate Prediction Center [38] Merged Analysis of Precipitation [39], while for 1961–1978 data from the Climate Research Unit of the University of East Anglia [40] is used.

Output from a total of nine atmospheric circulation models were used in this study: the National Aeronautics and Space Administration, Center for Ocean-Land-Atmosphere Studies, Geophysical Fluid Dynamics Laboratory and Scripps models have an horizontal resolution of ~ 2.0°, while the European Center for Medium Range Weather Forecasts model and National Center for Atmospheric Research Community Climate Model have an horizontal resolution of ~ 2.8°. With this set of models, retrospective probabilistic forecasts were produced using total of 144 members forced by evolving sea-surface temperatures, and 68 members forced by persisted sea-surface temperatures. For additional details see Table 2 in Barnston et al. [37].

**Table 2:** Potential utility of weather and climate predictions for vector borne disease control

Time frame	Climate driver	Availability for operational use	How forecast may be used in vector control
Weather forecasts	Numerical weather predictions provide the most robust short term weather forecasts.	In Africa few countries have capacities to skillfully predict the weather beyond 2 days. Extending such forecasts to 5 or even 10 days may be possible in some areas. Global weather forecasts are often poorly calibrated for local use.	Short term weather forecasts give little additional time for a vector-borne disease early warning system although they might provide valuable information on extreme events that may impact the health system more broadly.
Sub- Seasonal weather forecast (S2S)	The Madden-Julian Oscillation (MJO) is the dominant mode of sub-seasonal climate variability in the global tropics and a driver of predictability in S2S forecasts.	S2S experimental forecasts are becoming available from global producers.	S2S forecasts have yet to be shown as operationally useful for vector-borne disease control.
Seasonal climate forecasts	Slow changes in sea surface temperature (eg. equatorial Pacific ENSO events).	Operational seasonal forecasts are available from national, regional and global producrers. They are useful for predictable regions (Sahel, JAS), Eastern Africa, OND) and Southern Africa, DJF) - highest skill during ENSO periods.	When predictability is high seasonal climate forecasts can add months to early warning system that are already developed based on monitored rainfall and temperature.
Transition time scale forecasts (1–9 years)	Transition time scale between seasonal prediction and decadal variability.		Forecasts that are longer than seasonal climate forecast are highly desirable for planning purposes. However they are not operationally available for Africa.
Decadal forecasts (10–30 years)	Decadal SSTs for example SST variations over the Pacific Ocean are highly correlated with decadal rainfall variations in Eastern Africa March–May season.	Experimental forecasts only.	Decadal predictions are at the forefront of climate research but operational forecasts may not be realistic any time soon. However, where decadal variations are limited temperature may follow long term climate change trend.
Climate change scenarios	Long term changes in athropogenic gas emissions.	IPCC scenarios for global and regional scale. - regions where models agree. Downscaling of climate change scenarios is essential to relate this information to national and subnational decision-making.	Climate change scenarios provides some strong indication of long term warming trend but largely outside of operational vector-borne disease decision-time frames. Where the time line is relevant, e.g. in assessing climate risks to malaria eradication, rainfall scenarios are highly uncertain. Temperature trends, especially in the absence of strong decadal variability, may provide valuable information.

*S2S* Sub-seasonal to seasonal, *ENSO* El Niño Southern Oscillation, *JAS* July–August-September, *OND* October–November-December, *DJF* December–January-February, *SST* sea surface temperatures

## Results

The results from the analyses described above are all presented using the IRI Data Library Maproom capability and can therefore be explored directly by any interested reader (Table 1 for links).

### Analysis 1. Multi-timescale climate decomposition

**Fig. 4:**
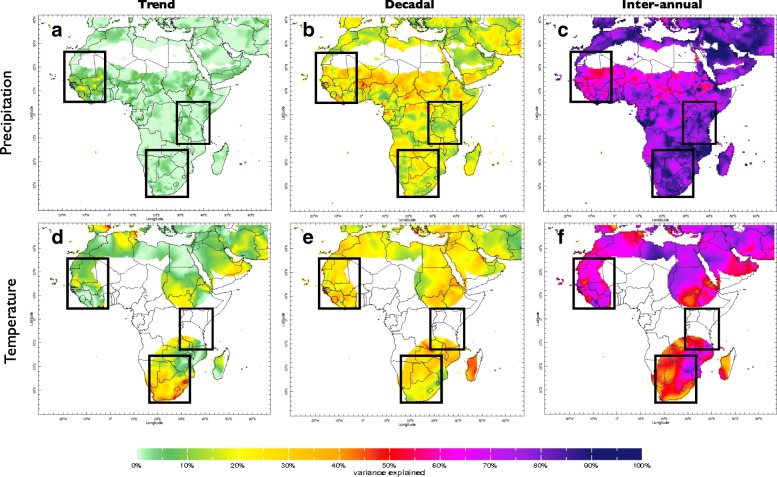
**a**–**f** Climate timescale decomposition for rainfall a,b&c and temperature d,e&f across Africa. Boxes indicate source of time series analysis for Western, Eastern and Southern Africa for Fig. 5a–f

**Fig. 5:**
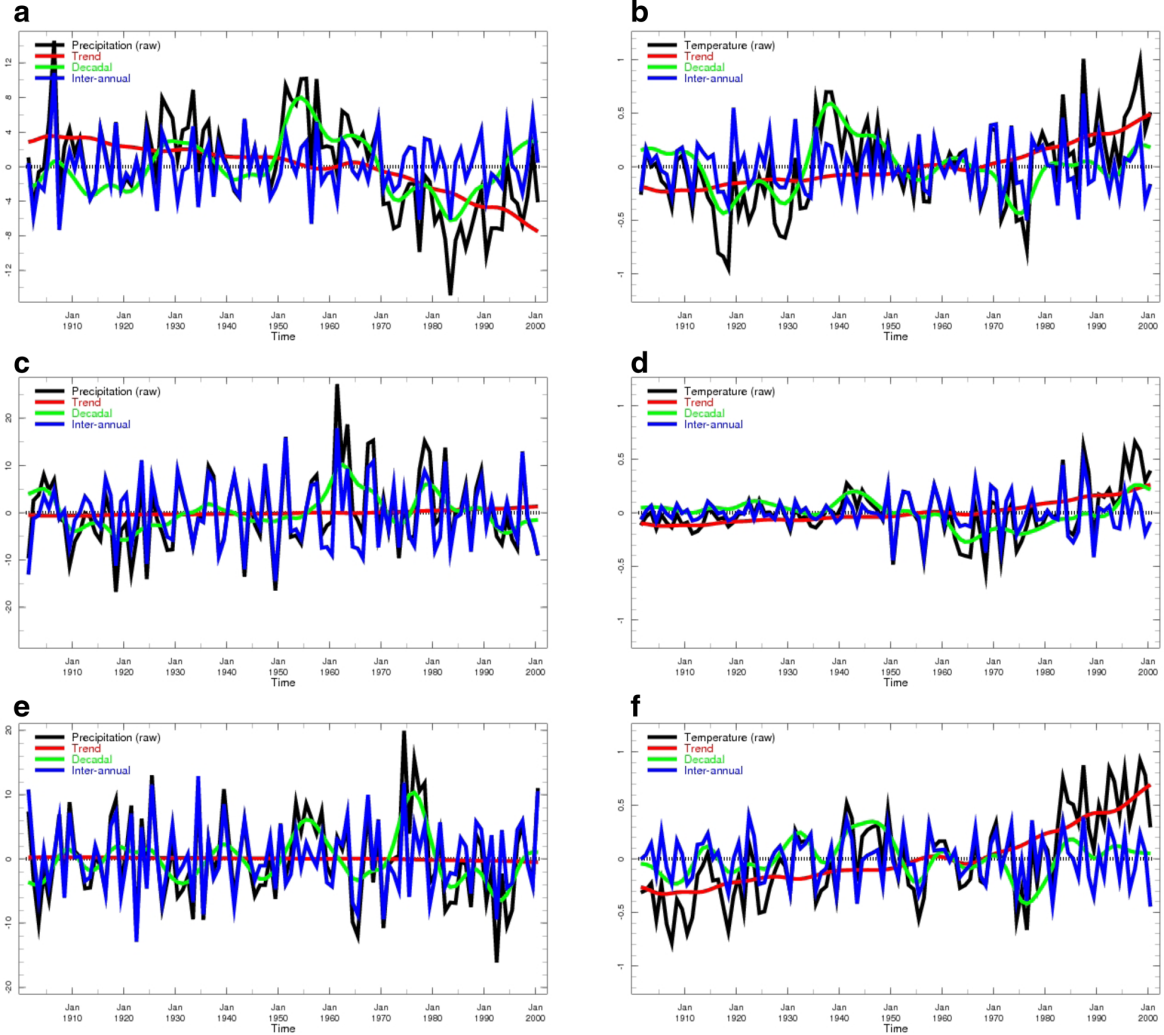
**a**–**f** Climate timescale decomposition for rainfall and temperature in Western (a&b) Eastern (c&d) and Southern Africa (e&f) with analysis averaged over boxed areas identified in Fig. 4a–f

The results of the timescale decomposition analysis for rainfall and temperature are presented in Figs. 4 and 5. Note that while the decomposition of a signal into trend, low- and high-frequency components may seem straightforward, the analysis presented involves a number of subtleties that are described in detail in documentation that can be found on the timescale decomposition Maproom site (see Table 1). The documentation also offers a number of caveats regarding the interpretation of Maproom displays.

#### Rainfall

The dominant source of variability in rainfall across the continent comes from the inter-annual timescale. Significant decadal variability also exists – especially across the Sahel region including Mauritania. There is minimal evidence of long term trends in rainfall across the continent using the University of East Anglia gridded rainfall data set.

#### Temperature

The UEA temperature data set has far fewer observations than for rainfall and consequently the poor quality of the century long, continent wide, data set limits the areas where robust analysis can be undertaken. However, despite these limitations it can clearly be seen that long term trends, decadal shifts and short-term variability in temperature all contribute to the observed variations in temperature across the three regions where the WHO-TDR consortium projects study sites are based.

### Analysis 2. Assessing the impact of the ENSO on rainfall and temperature across Africa

**Fig. 6:**
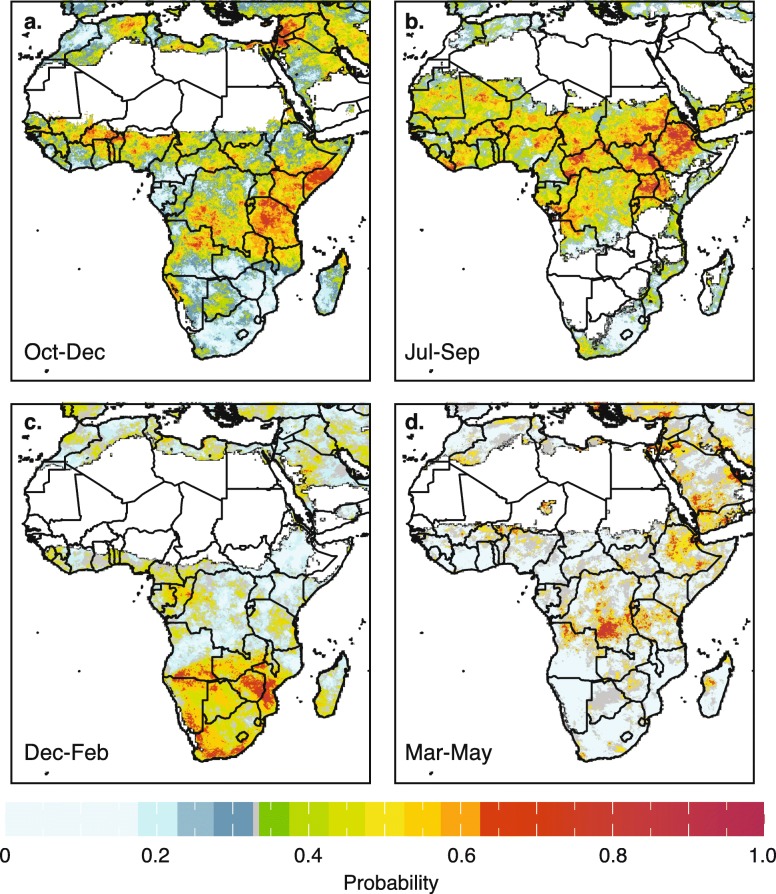
**a**–**d** These maps show the historical probability (given in percentile) of seasonal average of CHIRPS monthly rainfall falling within the upper (wet), one-third (“tercile”) of the 1983–2015 distribution in the country given the occurrence of El Niño/La Niña during that same season. A dry mask is used whenever the sum total of rainfall is ≤10 mm for the three month period. a) the probability of El Niño associated above normal rainfall for Oct–Dec (note the severe impact in Eastern Equatorial Africa); and b) El Niño associated below normal rainfall impact for Jul–Sep (note the severe impact in Ethiopia); c) La Niña associated above normal rainfall for Dec–Feb (note the severe impact in Southern Africa; d) El Niño associated above normal rainfall for Mar–May (note the absence of impact for this main rainy season in Eastern Africa

The positive and negative impact of the El Niño on rainfall in the October to December for Eastern Africa and July to September seasons (for the Sahel) respectively are presented in Fig. 6a&b, while Fig. 6c indicates the positive impact of La Niña conditions on the rainfall of Southern Africa during the main season (December to February). On the other hand, Fig. 6d shows no impact of El Niño on the main rainy season (March to May) in Eastern Africa. Additional analyses for other seasons and for temperature can be obtained directly from the Maproom (Table 1).

**Fig. 7:**
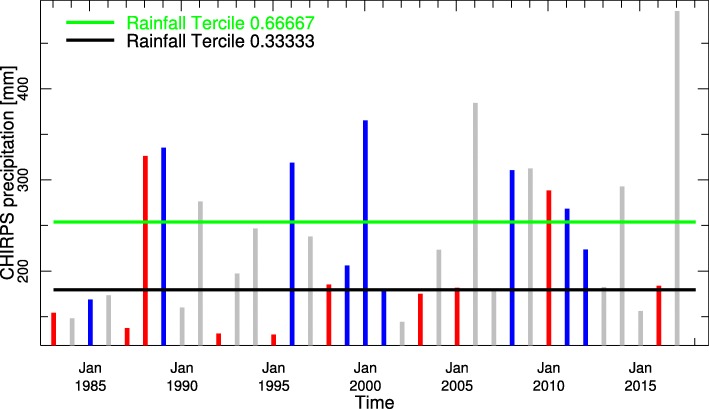
Spatially averaged yearly seasonal rainfall (Dec–Feb) time series for Botswana using CHIRPS (1982–2017). The color of the bars depicts the El Niño Southern Oscillation phase of the year, and the horizontal lines show the historical terciles limits. Note that 11/13 El Niño years (red) [41] have rainfall amounts within the normal to below normal range whereas 7/9 La Niña years (blue) have rainfall amounts predominantly within the normal to above normal range. Grey bars are for neutral years

The relationship of ENSO states to seasonal rainfall totals and average annual temperature time series are presented for Botswana in Fig. 7. The colour bars indicate the ENSO phase for an individual year, and the horizontal lines show the historical tercile limits. The image allows a quick assessment of the historical impact of ENSO by region and season and gives a visual indicator of the spread of results.

Note that the ENSO Maproom does not provide a forecast, but is a good tool for exploring the effect of different ENSO phases on seasonal rainfall and temperature. It is based on historical observations of rainfall and SST alone. Where a strong signal is found, it suggests that there is an opportunity for skillful seasonal forecasts since such forecasts substantially rely on a strong ENSO signal.

### Analysis 3. Assessing the impact of the ENSO on rainfall and temperature in Tanzania

**Fig. 8:**
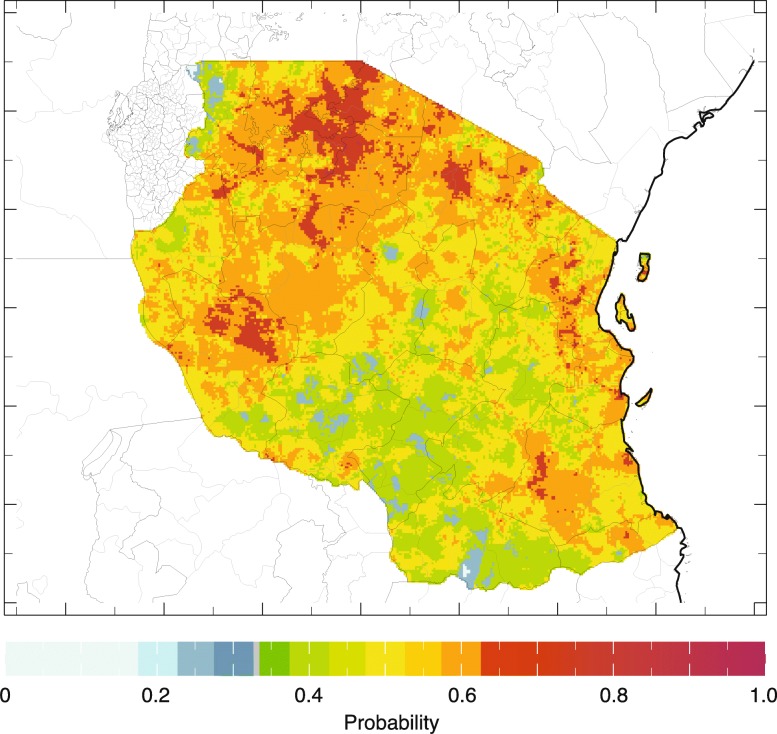
This map of Tanzania shows the historical probability of seasonal average monthly rainfall falling within the upper (wet) one-third (“tercile”) of the 1983–2010 historical distribution in the country given the occurrence of El Niño during that same season. The image depicts the probability of rainfall being above normal for the October–December season

**Fig. 9:**
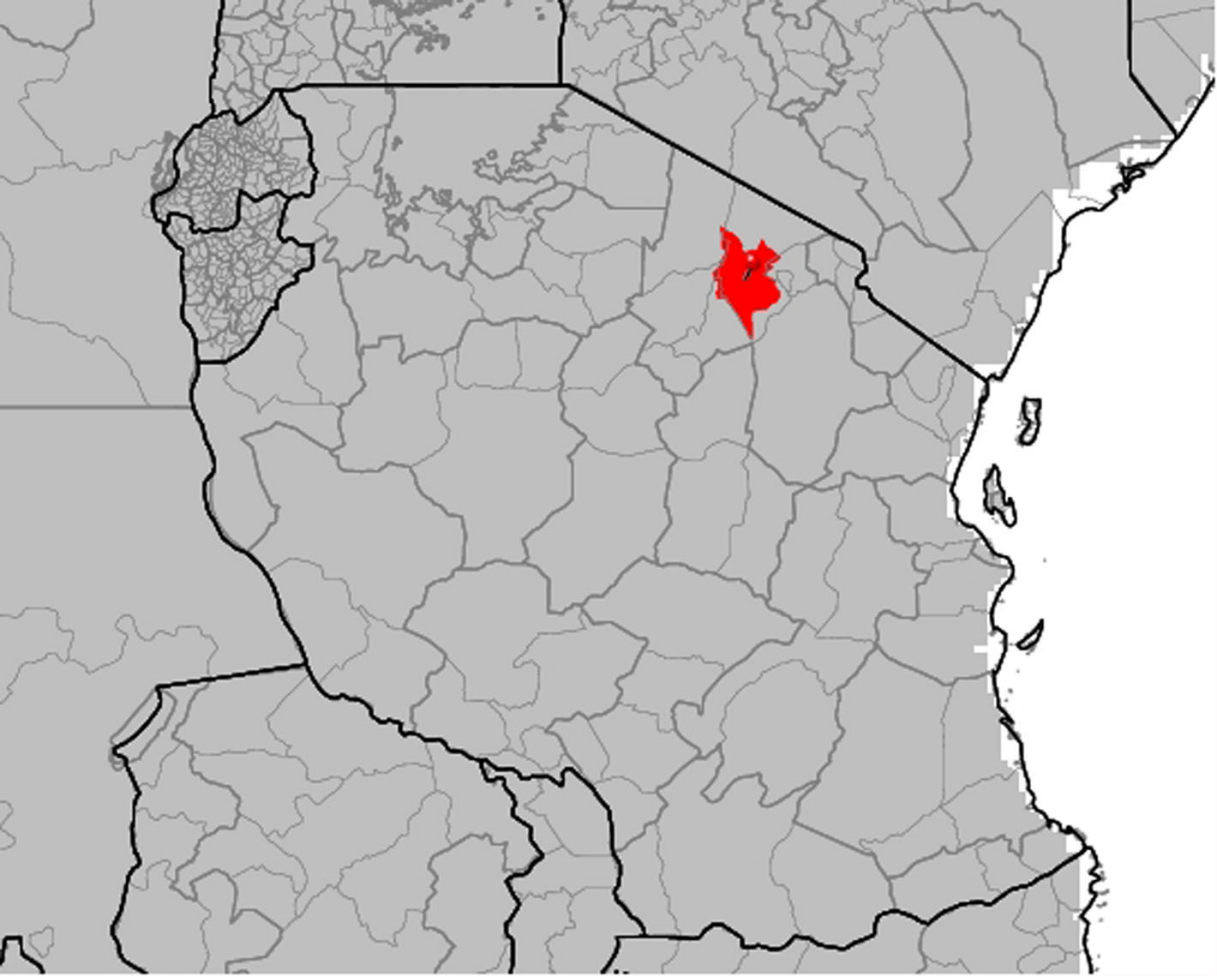
The geographic location of Monduli district, Arusha, Tanzania

**Fig. 10:**
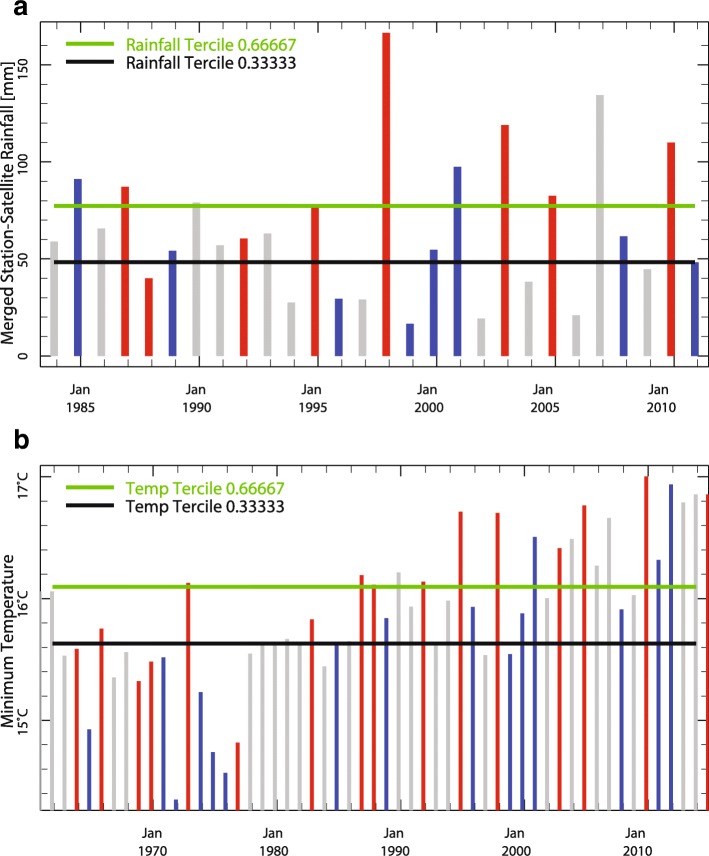
**a** & **b** Spatially averaged yearly seasonal rainfall time series for, Monduli, Tanzania using ENACTS climate products (1983–2014) for the October–December Season. The color of the bars depicts the ENSO phase of the year (El Niño red; La Niña blue bar; neutral grey) and the horizontal lines show the historical terciles limits; a) rainfall and b) minimum temperature. Note that El Niño years tend to be wet and warm relative to La Niña years

The results of the Pan-African ENSO analysis above were repeated in a national scale analysis using ENACTS products and services made available by the Tanzanian Meteorological Agency on their website (Table 1). The analysis indicates a moderate to strong impact of El Niño across the country associated with the Oct–Dec short rains (Fig. 8). A detailed analysis of the ENSO rainfall and temperature interaction for Monduli District, Tanzania (Fig. 9) for October–November-December (OND) is presented in Fig. 10a&b. Figure 10a indicates that El Niño years [41] have rainfall amounts predominantly within the normal to above normal range whereas La Niña years (blue) have rainfall amounts predominantly within the normal to below normal range. Figure 10b indicates that El Niño years [41] have minimum temperatures that are predominantly within the normal to above normal range whereas La Niña years (blue) have minimum temperatures predominantly within the normal to below normal range. Similar analysis indicting the correlation of the positive and negative phases of the Indian Ocean Dipole where completed using the Tanzanian Meteorological Agency (TMA) Maproom (not shown here). The same analysis can be done for Kenya and other ENACTS countries.

### Analysis 4. Assessing the predictability of seasonal rainfall and temperature across Africa

**Fig. 11:**
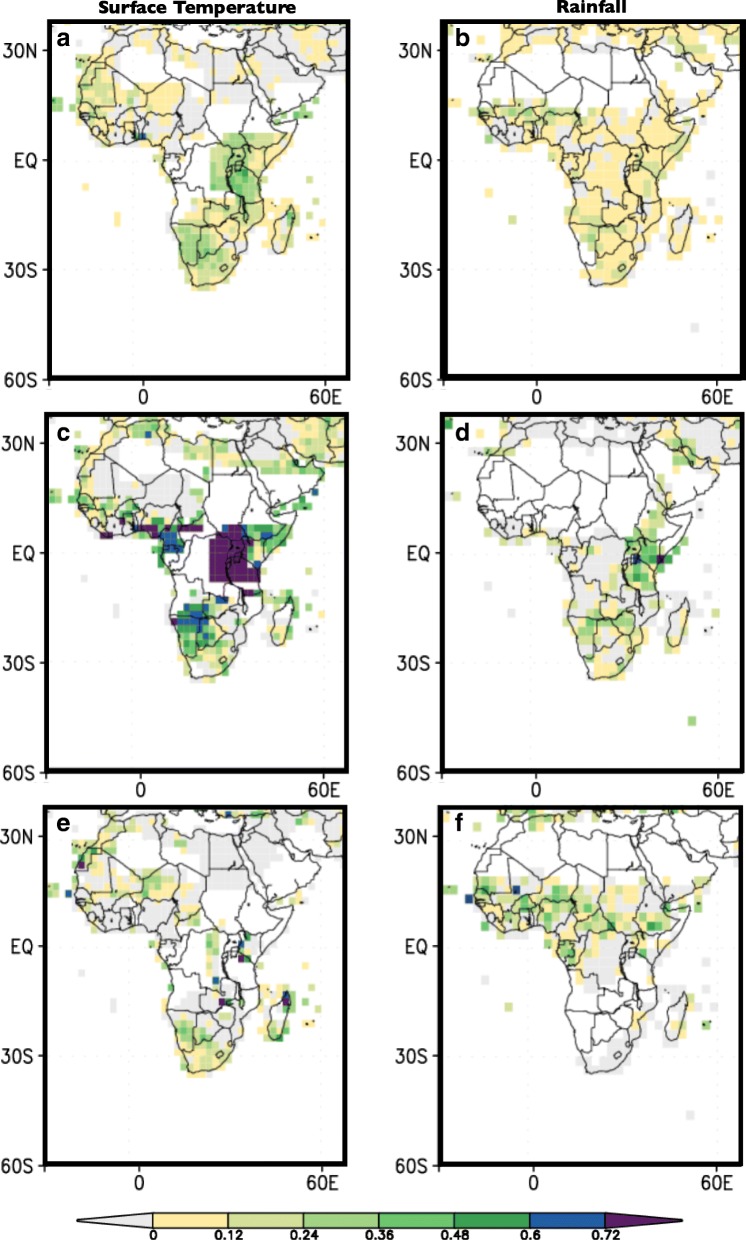
Forecast skill as measured by the Generalized Relative Operating Characteristics (GROC) metric, for the African continent. Surface temperature is shown on the left column, and rainfall is on the right. (**a**&**b**) All seasons, (**c**&**d**) Dec–Jan–Feb, (**e**&**f**) Jul–Aug–Sep. Lead time is 0.5 months

The skill of seasonal climate forecast across Africa, as measure by *the Generalized Relative Operating Characteristics (GROC) metric,* for temperature and rainfall forecasts averaged over the entire year is poor (see Fig. 11a&b). However, both temperature and rainfall seasonal forecasts demonstrate skill in certain regions when particular seasons are considered. For example, during DJF, temperature forecasts tend to be good in southern Africa where they coincide with the main rainy season and also in parts of western Africa. They are also skillful in eastern Africa for both rainfall and temperature despite the short rainy season being largely confined to OND (see Fig. 11c). Rainfall in the Sahel exhibits some predictability during the main July–August-September (JAS) season. Although it is not very high, the skill of forecasts for rainfall for this season is on average higher than surface temperature skill (see Fig. 11e&f). Note that the crude nature of the climate data used in the analysis will limit the evidence of predictability.

A summary of the predictability of the climate drivers (ENSO, Decadal, Long Term Change) over the climate of the WHO TDR study regions is provided in Table 3.

**Table 3:** Climate drivers and levels of predictability for WHO-TDR study regions + provides an indication of the strength of the relationship

Region	ENSO impact	ENSO predictability	Decadal Impact	Decadal predictability	Long term change	Climate Change predictions
Eastern Africa	+++ for rainfall for OND. +++ for temperature in tropics for extended period following ENSO onset. Tanzania - rainfall impact focused on northern and western regions Kenya - with temperature signal particular important in extensive highland areas.	+++ for rainfall OND in conjunction with Indian Ocean Dipole. +++ for temperature in tropics for extended period following ENSO onset. Tanzania - with rainfall forecast skill focused on regions where OND rainfall occurs. Kenya - with temperature prediction particular important in extensive highland areas.	+++ for rainfall for MAM. +++ for temperature.	Rainfall not predictable in operational context. + Temperature predictable from long term change if decadal signal is weak.	+++ for temperature warming. + Rainfall scenarios indicate wet.	+++ for temperatures warming. + for rainfall getting wetter.
Western Africa (including Sahel) (Valid for Mauretania and Ivory Coast)	++ for rainfall for JAS in Sahel. +++ for temperature in tropics for extended period following ENSO onset.	++ for rainfall for JAS in Sahel. +++ for temperature following ENSO onset.	+++ rainfall JAS. +++ temperature.	Rainfall not predictable in operational context. + Temperature not predictable in operational but long term change signal may be visible at shorter timescales if not masked by decadal signal.	+++ for temperature warming. Rainfall scenarios indicate both wet and dry.	+++ for temperatures warming. Rainfall highly uncertainty.
Southern Africa (Valid for Botswana and Zimbabwe)	+++ for rainfall in DJF. +++ for temperature in tropics for extended period following ENSO onset.	+++ for rainfall in NDJ. +++ for temperature in tropics for extended period following ENSO onset.	++ for rainfall season. +++ for temperature in tropics.	Rainfall not predictable in operational context. + Temperature long term change signal may be visible at shorter timescales if not masked by decadal signal.	+++ for temperature warming. ++ Rainfall observations indicate dry.	+++ for temperatures getting warming. ++ for rainfall drying.

+ = weak; ++ moderate; +++ strong; *ENSO* El Niño Southern Oscillation, *MAM* March–April-May, *JAS* July–August-September, *OND* October–November-December, *NDJ* November–December-January, *DJF* December–January-February

## Discussion

### Climate information into national decision-making for vector control purposes

Forecasting vector-borne diseases, such as malaria, using climate information is not new. Over a century ago records of unusual rainfall along with impoverished foods stocks were used as indicators of forthcoming malaria epidemics in the Punjab region of India [42]. In recent years an extensive research literature has emerged on the predictive relationship of observed and forecasted climate events in Africa and the spatial, seasonal, year to year and longer term shifts in vector-borne diseases [1]. Furthermore there has been an increase in studies providing evidence of the skillfulness of vector-borne disease forecasts based on climate monitoring products and seasonal climate predictions [14, 43, 44] and a greater interest in such analysis by policy-makers [4].

However, the promise of skillful and useful climate based early warning systems in Africa has been slow to materialize in practice. This is in part because:

1. Climate and disease mechanisms and relationships are often poorly understood and may not be consistent across space or time;
2. Seasonal climate forecasts are not universally applicable and should only be used when and where they are shown to be skillful. Because ENSO is a major source of predictability of the African climate forecasts have the greatest predictability during ENSO years, and in certain regions and seasons;
3. Concomitant disease and climate data of sufficient quality, historic length and appropriate spatial scale and coverage for development of evidence are needed to develop robust analysis but are not readily available;
4. Where data is available research may not be translatable to local operational systems; for example, if a forecast system is developed using historical data, such as reanalysis, which is not updated in real time, the research results will not translate into an operational system where near real time data is needed.
5. Where research results could technically translate to operational systems, institutional relationships, data policy issues, resources and capacity gaps may limit the development operationalization, and sustainability of Early Warning Systems.

A key challenge to accurately using climate information for vector-borne disease prediction is the spatial and temporal variability in climate variables of interest. While a range of variables may be relevant to transmission they may not be available for use in operational systems which require national coverage, relevance at the local scale and near real time updates. Temperature and precipitation conditions may be predictable in one region or season but this does not necessarily mean that it can be extended to another. The series of analysis presented here are designed to establish which timeframes of variability are most important and reliable for disease prediction in the different study regions.

### Analysis 1. Multi-timescale climate decomposition

The timescale decomposition analysis revealed that while century long term changes in rainfall were not a major historical concern across Sub-Saharan Africa during the twentieth century, decadal-scale variability has significant impacts on the climate, and hence populations and economies, in strongly affected areas such as the Sahel. This region shows the most extreme variations of seasonal climate anywhere in the world. Dramatic year to year variability in rainfall (in part related to ENSO events) is super-imposed upon decadal shifts in the climate as well as a long term drying trend. However, climate change models are uncertain as to the sign (wetter or drier), let alone the magnitude of potential changes in rainfall in this region. The decadal fluctuation in West African rainfall observed in Fig. 5b has been linked, in other studies, to SST variations in the Atlantic Ocean although the Indian Ocean may also be playing a role [20]. The long decline in rainfall during the 1970s and 1980s in the Sahel contributed to the retreat of malaria in this region [45]. The return to a higher rainfall regime in the last two decades (also likely a decadal variation) may have contributed to the re-emergence of *Anopheles funestus* to some areas, including Niger, after an absence of many years [46].

In East Africa, there has been a significant drying in the climate over the last two decades (Fig. 5c). This has occurred at a time when climate change models project that East Africa is getting wetter in the future – a phenomena called the “East African Climate Paradox” [22]. According to Lyon, the observed drying started abruptly in 1998 with a steep decline in the long rains (MAM) and is found to be driven strongly (although not necessarily exclusively) by natural decadal variability in the tropical Pacific rather than anthropogenic climate change [47]. The East African short rains (OND) are not affected by this decadal process further indicating distinct nature of these two seasons. As March–May is the main rainy season throughout much of Eastern Africa a dramatic decline in rainfall amounts in this season is likely to have a profound effect on vector-borne diseases such as malaria in affected areas [48].

There is also evidence of decadal variability in rainfall in Southern Africa (Fig. 5e) which has a tendency to become wetter during decadal periods when the eastern Pacific Ocean is cooler than average [47]. Mason and Jury [49] suggest there may be some periodicity of decadal variations in the climate of South Africa having a dominant period of about 18 years.

Continued warming of the planet is the most certain feature of climate change models [50]. Warming trends over the last century (and in particular from the 1970s, is evident in all regions of Africa where data is sufficient for analysis (see Figs. 4d and 5bd,f). For instance there is now substantive evidence that the East African highland region has been warming over the last 30 years [19, 51] with potential impacts on malaria and other vector-borne disease transmission in areas where transmission has hitherto been constrained by low temperatures.

### Analysis 2: Assessing the impact of the ENSO on rainfall and temperature across Africa

Our results are consistent with what is known about ENSO and the climate of Eastern Africa. Here the annual cycle of rainfall tends to be bi-modal, with two physically and statistically uncorrelated rainy seasons [26] occurring in October–December (short rains) and March–May (long rains). Year-to-year variability of the short rains is frequently associated with ENSO [24]; but this connection depends critically on sea surface temperatures in the Indian Ocean, not just the Pacific. El Niño is typically associated with wetter than average conditions, while La Niña is frequently associated with drought in the short (OND) rainy season. A positive Indian Ocean Dipole (IOD) [52] is also associated with enhanced short rains; its opposite phase with drier than average conditions. While we have not undertaken an IOD analysis the relationship and can be explored in local East African ENACTS Maprooms (iri.columbia.edu/ENACTS).

Rainfall in many parts of the northwestern region of Eastern Africa (western Ethiopia and parts of western Kenya) have a boreal summer rainy season from June–September which is more in common with the timing of the Sahelian rainy season. The climate of the Sahel exhibits typical monsoon behavior, with a single peak in the rainy season between June–September. Our results support other studies which find a modest connection between ENSO and seasonal rainfall variability in the Sahel [53] with El Niño events associated with drier than average conditions and La Niña with wetter than average conditions.

Our results are also consistent with what we now about the climate of Southern Africa which is influenced by atmospheric circulations in both the tropics and the mid-latitudes. The main rainy season typically extends from October–April across much of the region, peaking during the southern-most extension of the inter-tropical convergence zone. By contrast, the southern tip of South Africa has a maximum in rainfall during the southern hemisphere winter season (May–September), associated primarily with the passage of mid-latitude storm systems [49]. A relationship between seasonal rainfall variability and ENSO has been observed in the region [54]. El Niño events are typically associated with drought in Southern Africa with La Niña linked to wetter than average conditions, although even strong El Niño events are not necessarily accompanied by drought [55]. There is substantive evidence that malaria in southern Africa is affected by SSTs in the Eastern Pacific (the Niño 3.4 region) with La Niña events frequently associated with an increased occurrence of cases [56, 57].

While we have not considered in detail the climate of Central Africa, we note that it contains the second largest area of tropical rainforest on earth and is therefore an important, but poorly studied, part of the global climate system [41]. It also has a high burden of malaria. The annual cycle of rainfall shows a bimodal behavior, with relative rainy seasons peaking in March–May and October–December, although there is substantial rainfall outside these seasons. The variability of the climate of Central Africa has received comparatively little attention compared to other parts of the continent [58]. On seasonal to inter-annual timescales, some studies have suggested a relationship between rainfall variability in Central Africa and SSTs in the tropical southern Atlantic Ocean [59]. For example, warmer than average SSTs off the Angolan coast are associated with increased rainfall, particularly in the March–May season and in the western part of the region. It should be noted that the quality of climate data for this region is extremely poor with few operational meteorological stations available. Consequently, global products for this region are likely also poor.

Our results (Fig. 5a, b) are consistent with other studies that show only a weak link between seasonal rainfall variability and ENSO in Central Africa with the largest connection found during the boreal fall season where El Niño (La Niña) events are associated with drier (wetter) than average conditions [60].

It is to be expected that the signal of the inter-annual relationship between climate and vector-borne diseases in Central Africa will also be weak as the environment is consistently warm and humid with high levels of rainfall throughout much of the year. Variations are likely insufficient to impact on transmission although there is scant vector or case data to establish whether or not this is the case.

### The value of high resolution climate data in assessing the impact of ENSO on rainfall and temperature at the subnational level

National climate datasets made available through the Enhancing National Climate Services (ENACTS) initiative, provide additional insights into the relationship of ENSO (and the Indian Ocean Dipole) to rainfall and temperature variations at spatial scales which are relevant for vector-borne disease monitoring and prediction. The higher quality data sets are created from a blend of all the relevant observations made available by the National Meteorological and Hydrological Services, with the best global products. The improved quality of the data sets over global products make it easier to reveal the predictability that exists. Similar analysis are now possible in all countries where ENACTS is being implemented (see Table 1).

### The relative importance of climate drivers and their potential predictability

The relative importance of the three categories of climate drivers and their predictability are region and variable specific. For year-to-year-variations, ENSO is the predominant driver of variability in rainfall and temperature and ENSO impacts on the climate can be observed most strongly during the single rainy seasons of Southern Africa and the Sahel and the short rains of Eastern Africa. Decadal variations in rainfall are also significant in the Sahel and have been observed for the March–April–May rainy season in Eastern Africa (not shown). Long term trends are observed the temperature data for southern and western Africa but the analysis for eastern Africa is constrained by data quality. Challenges encountered when seeking predictions at climate timescales are outlined in Table 2. In particular, our ability to assess forecast/prediction/scenario skill at different time scales is constrained by the lack of sufficiently long historical climate data. To observe the accuracy of a weather forecast one needs to wait a day or two and then the expired forecasts can be assessed against what is observed. Within a season there is plenty of data which can be used to assess forecast skill. For seasonal prediction, many regions only have one or at most two rainy seasons. Since seasons may act independently they each need to be treated in separate analysis. Thus assessing the skill of a probabilistic seasonal climate forecasts requires a minimum of 30 or more years of climate data against which the forecast models can be run in “hindcast mode”. Seasonal climate forecasts (both rainfall and temperature) are predicted shifts in the probability density function of seasonal rainfall totals or temperature means relative to a climatological baseline. The forecasts are commonly expressed in tercile probability format (i.e., probabilities of below-normal [BN], near-normal [NN] and above-normal [AN] rainfall or temperature categories). Thus, within a pdf of 30 years of climate data we have 10 years BN, 10 years NN and 10 years AN. With this short time series signals have to be very strong to be statistically significant. Describing a year as above-normal, provides little indication of the likely outcome in terms of disease. Is the season likely to be extremely wet? above a certain rainfall threshold? with rainfall events well distributed over time?. These types of questions are increasingly being addressed by climate scientists and we may expect much more nuanced seasonal forecasts to be available in the near future.

The quality of the data used to assess forecast skill also matters. If the data set gives a poor indication of actual climate conditions, then the skill test results are likely to be poor. Where available the ENACTS historical climate data (30+ years) provides a high quality climate data set for use as the forecast predictand.

The challenge of verifying forecasts that will happen decades into the future become even more onerous. As there are few places in the world where historical climate datasets go back sufficiently long in the past to assess variability over 10–30 year time frames, a more general validation of the model is needed. This is based on an understanding of its underlying mechanisms and the relationship of model outputs to historical climate characteristics of the region of interest. This is also true for the assessment of climate change model outputs.

## Conclusions

Climate varies across the African continent. These variations have the potential to significantly impact vector-borne disease dynamics at multiple space and time scales. Weather and climate information (past, present and future) may be used for operational vector programmes; their advantages and limitations are summarized below:

1. Historical observations of rainfall, temperature and humidity provide valuable information for understanding past variations in vector-borne disease if quality information is available at the space and time scales of the vector/health data (for example, ENACTS-implementing countries).

2) Recent and current observations of rainfall and temperature (and humidity when available) provide a significant resource for predicting changes in vector-borne diseases months ahead of time if quality information is available at relevant space time scales and in near-real time.

3) Weather forecasts provide limited advanced notice (only a few days at best) of epidemics above what is available from rainfall and temperature monitoring information.

4) Sub-seasonal climate forecasts are an area of significant research and, while not very skillful, may help bridge the gap between weather and seasonal forecasts in some areas.

5) ENSO impacts on rainfall on the African continent are observed predominantly in Eastern and Southern Africa with a more moderate impact in the Sahel. Predictions of ENSO state (El Niño, Neutral and La Niña) can provide some limited early warning of drought or wetter conditions in some regions and seasons.

6) Seasonal climate forecasts, available from Regional Climate Centers or National Meteorological Agencies, which integrate ENSO state and other predictors, are likely to be most useful as a component of early-warning systems for vector-borne diseases. This assessment is expected to be especially true for the single rainy season in Southern Africa (December–February), and for the short rains (October–December) in Eastern Africa, where they are most skillful.

7) Decadal variations in climate are significant in some regions (e.g. the Sahel) and seasons (e.g. March–May in Eastern Africa). Decadal variations can impact the perception or expectations of anthropogenic climate change, as short-term shifts in the climate (10–30 year) are easily confused with longer-term trends. Decadal climate prediction is in its infancy and it is not certain that skillful forecasts will emerge that can be used operationally.

8) Long-term trends in warming are most likely to have the greatest impact in the highland areas of Eastern and Southern Africa where current temperatures restrict the development rates of vectors and pathogens. Climate change projections may provide relevant information on long term trends (e.g. for 2080 and beyond), but these are commonly too far into the future to be use of use to policy makers concerned with considerations of disease control. In the absence of significant decadal variations long-term trends can be used to provide a strong indication of likely trends at shorter time scales, e.g., the next few decades.

Given the above, EWS for vector-borne diseases should be developed using an integration of historical knowledge, current climate context as well as skillful operational seasonal climate forecasts.

## Additional file

Additional file 1: Multilingual abstracts in the six official working languages of the United Nations. (PDF 577 kb)

## Abbreviations

CRU: Climate Research Unit of the University of East Anglia
DJF: December–January-February
ENACTS: Enhancing National Climate Services
ENSO: El Niño Southern Oscillation
ERSST: Extended reconstructed sea surface temperature
EWS: Early warning systems
GPCC: Global Precipitation Climatology Center
GROC: Generalized Relative Operating Characteristics
IOD: Indian Ocean Dipole
IRI: International Research Institute for Climate and Society
JAS: July–August-September
JJA: June–July-August
MAM: March–April-May
NCDC: National Climate Data Center
NOAA: National Oceanic and Atmospheric Administration
OND: October–November-December
ONI: Oceanic Niño Index
S2S: sub-seasonal to seasonal
SST: sea surface temperature
TDR: Tropical Disease Research
WHO: World Health Organization
WMO: World Meteorological Organization
