# Supplementary material for: Climate drivers of vector-borne diseases in Africa and their relevance to control programmes
Source: Infect Dis Poverty. 2018 Aug 10;7:81. doi: 10.1186/s40249-018-0460-1 (PMC6085673; doi:10.1186/s40249-018-0460-1)
Supplement: Supplementary file 1 — Multilingual abstracts in the six official working languages of the United Nations. (PDF 577 kb) [file 40249_2018_460_MOESM1_ESM.pdf]

Translation of the abstract into the six official working languages of the United Nations

العوامل المناخية الخاصة بالأمراض المنقولة عبر الحشرات في إفريقيا وصلتها ببرامج التحكم

مادلين س.ثومسن ,أنجيل ج. مونيوز , ريمي كزن و جوي شوميك غليموت

الملخص

خلفية: تم اقتراح التنبؤ بالأمراض المعتمدة على المناخ كأداة محتملة للتكيف مع تغير المناخ في القطاع الصحي. نستكشف هنا مدى ملائمة البيانات المناخية والعوامل والتنبؤات لجهود مكافحة الأمراض المنقولة عبر الحشرات في أفريقيا.

الطرق: باستخدام بيانات من عدد من المصادر، نستكشف هطول الأمطار ودرجات الحرارة في جميع أنحاء القارة الأفريقية، بدءًا من الفصول الموسمية وانتهاءً بالتغيرات على أساس سنوي ومتعدد العقود والمجال الزمني المصاحب لتغير المناخ. نولي اهتماماً خاصاً لثلاث مناطق محددة كمناطق الدراسة التابعة لمنظمة الصحة العالمية في غرب أفريقيا وشرقها وجنوبها. وتشمل تحليلاتنا 1) تحليل النطاق الزمني لتحديد الأهمية النسبية للاتجاهات على أساس سنوي، وأساس عقدي وطويل الأمد لهطول الأمطار ودرجة الحرارة ؛ 2) تأثير التذبذب الجنوبي (ENSO) على هطول الأمطار ودرجة الحرارة على عموم نطاق أفريقيا. 3) تأثير ظاهرة التذبذب الجنوبي في مناخ تنزانيا باستخدام منتجات مناخية عالية الدقة و 4) إمكانية التنبؤ المحتملة للمناخ في مختلف المناطق والمواسم باستخدام خصائص التشغيل النسبي المعممة. نستخدم هذه التحليلات لمراجعة مدى أهمية التنبؤات المناخية لاستعمالها في مكافحة الأمراض المنقولة عبر الحشرات عبر القارة.

النتائج: كشف تحليلُ النطاق الزمني عن الدفء طويل الأمد في جميع المناطق الثلاث في أفريقيا - عند مستوى 0.1-0.3 درجة مئوية في كل عقد. كانت الاختلافات العقدية في هطول الأمطار واضحة في جميع المناطق، وكانت واضحة بشكل خاص في منطقة الساحل وأثناء هطول الأمطار الطويلة في شرق إفريقيا (مارس - مايو). وكان الاختلاف من سنة إلى أخرى في كل من هطول الأمطار ودرجة الحرارة المرتبطة جزئياً بظاهرة التذبذب الجنوبي، بمثابة الإشارة السائدة للتغيرات المناخية على أي نطاق زمني. تم تحديد البيانات المناخية المرصودة والتنبؤات المناخية الموسمية باعتبارها المصدر الأكثر ملاءمة للمعلومات المناخية لاستخدامها في أنظمة الإنذار المبكر للأمراض المنقولة عبر الحشرات، ولكن هذه الأخيرة تختلف في المهارة حسب المنطقة والموسم.

الاستنتاجات: يعد التكيف مع مخاطر الأمراض التي تنقلها ناقلات الأمراض وتقلب المناخ وتغيره من الأولويات بالنسبة للحكومة والمجتمع المدني في البلدان الأفريقية. إن فهم تغيرات واتجاهات سقوط الأمطار والحرارة في جداول زمنية متعددة وقابلية التنبؤات المحتملة هي خطوة أولى ضرورية لدمج المعلومات المناخية ذات الصلة في عملية صنع القرار لمكافحة الأمراض المنقولة عن طريق ناقلات الأمراض.

Translated from English version into Arabic by Rand Gharaibeh, proofread by Aya AlAjjan, through

TRANSLATORS WITHOUT BORDERS

非洲媒传疾病的气候驱动因素及其与防治规划的相关性

Madeleine C. Thomson, Ángel G. Muñoz, Remi Cousin and Joy Shumake-Guillemot

摘要

引言：基于气候的疾病预测已被提出可作为卫生部门适应气候变化的潜在工具。本研究探讨了气候数据、驱动因素与预测非洲媒传疾病防治成效之间的相关性。

方法：利用来自多个来源的数据，我们对整个非洲大陆长时间跨度（从季节到年度和年代际）与气候变化一致的降雨和气温数据进行研究。特别关注西非、东非和南非这三个作为 WHO-TDR 研究区的地区。本研究包括：1）以时间尺度评估不同时间跨度（逐年、年代际和长期）降雨和气温变化趋势的相对重要性；2）厄尔尼诺南方涛动（ENSO）对泛非洲地区降雨和温度的影响； 3）ENSO 对采用高分辨率气候设备的坦桑尼亚的影响，以及 4）采用 GROC 对不同地区和季节的气候潜在预测性的影响。我们利用上述分析来审查气候预测对整个非洲大陆媒传疾病防治的影响。

结果: 分解时间尺度显示非洲三个地区长期变暖——每十年升高 0.1–0.3 ℃。降雨的年代际变化在所有地区都很明显，尤其是萨赫勒地区和东非的雨季(3–5 月)。降雨量和温度的年变化(部分与 ENSO 有关)是任何时间尺度上气候变化的主要信号。观测到的气候数据和季节气候预测被确定为媒传疾病预警系统最相关的信息来源，但后者在技术上因地区和季节而异。

结论：适应气候变化引起的媒传疾病传播风险是非洲国家政府和民间社会的首要任务。了解多个时间尺度下降雨和温度变化及趋势以及其潜在的可预测性，是将相关气候信息纳入制定媒传疾病防治决策的首要步骤。

Translated from English version into Chinese by Peng Song, edited by Pin Yang

CHINESE CENTER FOR DISEASE CONTROL AND PREVENTION
NATIONAL INSTITUTE OF PARASITIC DISEASES

Les facteurs climatiques des maladies à transmission vectorielle en Afrique et leur pertinence pour les programmes de lutte

Madeleine C. Thomson, Ángel G. Muñoz, Remi Cousin et Joy Shumake-Guillemot

Résumé

Contexte: La prévision des maladies d'origine climatique a été proposée comme un outil potentiel d'adaptation au changement climatique pour le secteur de la santé. Ici, nous examinons la pertinence des données, des facteurs et des prédictions climatiques dans la lutte contre les maladies à transmission vectorielle en Afrique.

Méthodes: En utilisant des données provenant de diverses sources, nous étudions les précipitations et les températures à travers le continent africain, de la saisonnalité à la variabilité à des échelles annuelle et multi-décennale, compatibles avec le changement climatique. Nous nous penchons surtout sur le cas de trois régions définies comme zones d'étude WHO-TDR: l'Afrique de l'Ouest, l'Afrique de l'Est et l'Afrique australe. Nos analyses incluent 1) une décomposition de l'échelle des temps pour établir l'importance relative des tendances annuelles, décennales et à long terme des précipitations et des températures; 2) l'impact de l'oscillation Australe El Niño (ENSO) sur les précipitations et les températures à l'échelle panafricaine; 3) l'impact d'ENSO sur le climat de la Tanzanie avec l'utilisation de produits climatiques à haute résolution et 4) la potentielle prévisibilité du climat en différentes régions et saisons, en utilisant des caractéristiques de fonctionnement relatives généralisées. Nous utilisons ces analyses pour examiner la pertinence des prévisions climatiques en vue d'une application dans la lutte contre les maladies à transmission vectorielle à travers le continent.

Résultats: La décomposition de l'échelle de temps a révélé un réchauffement à long terme dans les trois régions d'Afrique - à raison de 0,1–0,3 °C par décennie. Des variations décennales des précipitations ont été observées dans toutes les régions, plus particulièrement au Sahel, pendant la grande saison des pluies en Afrique de l'Est (mars-mai). La variabilité des précipitations et des températures d'une année à l'autre, en partie associée à l'ENSO, était le signal dominant des variations climatiques à n'importe quelle échelle de temps. Les données climatiques observées et les prévisions climatiques saisonnières ont été identifiées comme les sources les plus pertinentes d'informations climatologiques à utiliser dans les systèmes d'alerte précoce pour les maladies à transmission vectorielle, mais ces dernières variaient selon les régions et les saisons.
Conclusions: L'adaptation aux risques de maladies à transmission vectorielle de la variabilité et du changement climatique est une priorité pour le gouvernement et la société civile dans les pays africains. Comprendre les variations et les tendances de la pluviométrie et de la température à des échelles de temps multiples, ainsi que leur prévisibilité potentielle, est une première étape nécessaire dans l'intégration des informations climatiques pertinentes dans la prise de décision sur le contrôle des maladies à transmission vectorielle.

Translated from English version into French by Paustelita, proofread by Louis Gauvreau, through

TRANSLATORS
WITHOUT BORDERS

Климатические стимулы трансмиссивных болезней в Африке и их актуальность для использования в программах контроля

Мадлен С. Томсон, Анхель Дж. Муньос, Реми Кузан и Джой Шумейк-Гильмо

Аннотация

Краткое описание: Мы предлагаем использовать в здравоохранении прогнозирование болезней, связанных с климатом как возможный инструмент адаптации к изменениям климата. В этом исследовании мы рассматриваем применение климатических данных, стимулирующих факторов и прогнозов в борьбе с трансмиссивными болезнями в Африке.
Методы: Количество осадков и температура на Африканском континенте проанализированы по нескольким источникам от сезонности до вариабельности по годичным и декадным временным шкалам с учётом климатических изменений. Мы уделили особое внимание трём регионам, выделенным как зоны исследования ВОЗ-ПТБ в Западной, Восточной и Южной Африке. Наш анализ включает 1) разложение по шкале времени для установления относительной важности тенденций в количестве осадков и температур по годам, десятилетиям и долгосрочным периодам; 2) влияние Южного колебания Эль-Ниньо (ENSO) на количество осадков и температуру по Панафриканской шкале; 3) влияние ENSO на климат Танзании с использованием климатических продуктов с высоким разрешением и 4) потенциальную предсказуемость климата для разных регионов и сезонов при помощи обобщённых

относительных оперативных характеристик. Мы использовали эти виды анализов, чтобы изучить актуальность климатических прогнозов для применения их в борьбе с трансмиссивными болезнями по всему континенту.

Результаты: Разложение по шкале времени выявило длительное потепление во всех трёх регионах Африки на уровне 0,1–0,3 °C за десятилетие. Были замечены очевидные изменения по десятилетиям в количестве осадков во всех регионах; они были особенно ярко выражены в Сахеле и в период затяжных дождей в Восточной Африке (март – май). Годичная вариабельность обоих показателей, частично связанная с ENSO, была доминирующим признаком климатических изменений по всем временным шкалам. Данные климатических наблюдений и сезонные климатические прогнозы показали себя наиболее подходящими источниками климатической информации для использования в системах раннего предупреждения трансмиссивных болезней, однако, успешность прогнозов зависит от региона и сезона.

Выводы: Адаптация к рискам трансмиссивных болезней климатической вариабельности и к изменениям является приоритетом для правительств и гражданских обществ африканских стран. Понимание изменений и тенденций количества осадков и температур по многим временным шкалам и их потенциальной предсказуемости послужит необходимым шагом к включению надлежащих климатических данных в принятие решений по борьбе с трансмиссивными болезнями.

Translated from English version into Russian by Natalia Potashnik, proofread by TatianaKary, through

TRANSLATORS WITHOUT BORDERS

Los factores climáticos en enfermedades transmitidas por vectores en África y su importancia en los programas de control

Madeleine C. Thomson, Ángel G. Muñoz, Remi Cousin y Joy Shumake-Guillemot

Resumen

Contexto: La previsión de enfermedades producidas por el clima se ha propuesto como una posible herramienta para la adaptación del sector sanitario al cambio climático. Aquí analizamos la importancia de los datos, factores y predicciones climáticos en los programas de control de enfermedades transmitidas por vectores en África.

Metodología: Basándonos en datos de varias fuentes, examinamos las precipitaciones y la temperatura en todo el continente africano, desde la estacionalidad hasta la variabilidad de forma anual, multidecenal y en períodos de tiempo que sean consistentes con el cambio climático. Prestamos especial atención a tres regiones que se consideran zonas de estudio en el Programa Especial para la Investigación y Capacitación en Enfermedades Tropicales (TDR) de la OMS en África occidental, oriental y meridional. Nuestros análisis incluyen 1) una escala temporal de descomposición para

establecer la importancia relativa de las tendencias de precipitación y temperatura de año a año, en una década y a largo plazo ; 2) el impacto de El Niño-Oscilación del Sur (ENOS) en las precipitaciones y en la temperatura a escala panafricana; 3) el impacto de ENOS en el clima de Tanzania mediante el uso de productos climáticos de alta resolución y 4) la posible predicción del clima en diferentes regiones y estaciones mediante el uso de Características Operativas Relativas Generalizadas. Utilizamos estos análisis para revisar la importancia de los pronósticos climáticos en la aplicación de programas de control de enfermedades transmitidas por vectores en todo el continente.

Resultados: La escala temporal de descomposición mostró un calentamiento a largo plazo en las tres regiones de África – a un nivel de 0,1 a 0,3 ℃ por década. Las variaciones decenales de las precipitaciones fueron evidentes en todas las regiones y se acentuaron de forma considerable en la región de El Sahel y durante la época de fuertes lluvias en África oriental (de marzo a mayo). La variabilidad de año a año tanto en las precipitaciones como en la temperatura, en parte asociada a ENOS, fue una señal predominante de las variaciones climáticas en cualquier período de tiempo. Los datos climáticos y las previsiones climáticas estacionales observados se identificaron como las fuentes más relevantes de información climática para su uso en sistemas de alerta temprana de las enfermedades transmitidas por vectores, pero la segunda presentó variaciones en capacidad por región y estación.

Conclusiones: Para el gobierno y la sociedad civil en los países africanos es una prioridad adaptarse a los riesgos que conllevan las enfermedades transmitidas por vectores que causan la variabilidad y el cambio climáticos. Entender las variaciones de las precipitaciones y de la temperatura, y las tendencias en períodos de tiempo múltiples y su posible predicción es un primer paso necesario para incorporar la información climática relevante en la toma de decisiones en el control de enfermedades transmitidas por vectores.

Translated from English version into Spanish by Mutis, proofread by Ekaterina Lunina, through

TRANSLATORS
WITHOUT BORDERS
